# High expression of class III β-tubulin in small cell lung carcinoma

**DOI:** 10.3892/ol.2013.1734

**Published:** 2013-12-06

**Authors:** STEVEN POWELL, ALEX KAIZER, JOSEPH S. KOOPMEINERS, CARLOS IWAMOTO, MARK KLEIN

**Affiliations:** 1 Division of Hematology, Oncology and Transplantation, Department of Medicine, University of Minnesota, Minneapolis, MN 55418, USA; 2 Hematology/Oncology Section, Primary Care Service Line, Minneapolis VA Healthcare System, Minneapolis, MN 55417, USA; 3 Division of Biostatistics, University of Minnesota School of Public Health, Minneapolis, MN 55418, USA; 4 Department of Pathology, Minneapolis VA Healthcare System, Minneapolis, MN 55417, USA

**Keywords:** class III β-tubulin, small cell lung carcinoma, microtubule inhibitor

## Abstract

Class III β-tubulin (TUBB3) is emerging as a biomarker in a number of cancers. TUBB3 has been shown to be a prognostic indicator of more aggressive disease and a predictor of resistance to taxanes and vinca alkaloids. To date, there is little data on TUBB3 expression in small cell lung carcinoma (SCLC). The primary objective of this study was to determine the expression of TUBB3 in SCLC. Immunohistochemical staining of SCLC tumor specimens was performed using standard procedures. Expression of TUBB3 was determined as a composite of the percentage of malignant cells staining positive and the intensity of staining. Clinical and tumor data for each patient was compared with the degree of TUBB3 expression. A total of 66 SCLCs were evaluable for TUBB3 expression. The majority of specimens (n=56, 85%) had high expression of TUBB3. Only 4.5% (n=3) had low expression of TUBB3. The mean distribution of positive staining for the specimens was 87.3±1.8% (mean ± SE). Specimens from core biopsies were significantly more likely to have high TUBB3 expression when compared with fine needle aspirates (P=0.004). There were no other significant findings when comparing clinical or tumor characteristics. Overall, we found that expression of TUBB3 in SCLC is higher than expected. Innate resistance to microtubule inhibitors, such as the taxanes and vinca alkaloids, may be associated with this finding. Attempts at microtubule inhibition with novel agents may be able to overcome this resistance mechanism. Further evaluation of TUBB3 as a biomarker in SCLC is warranted.

## Introduction

Small cell lung cancer (SCLC) accounts for ~15% of all lung cancer diagnoses in the United States. The histology is unique and the disease is commonly referred to as a high-grade neuroendocrine carcinoma. The cancer behaves differently than non-small cell carcinomas (NSCLCs), as it is typically more aggressive, with metastatic disease usually present at diagnosis (1). In addition, SCLC is characteristically initially very responsive to chemotherapy and/or radiation, with response rates in the 60–80% range (2). However, once it has relapsed, SCLC is rather resistant to chemotherapy. This resistance to therapy equates to a poor median survival time of ~9–11 months in patients treated for metastatic disease (3). Understanding both innate and acquired resistance in SCLC is key to developing improved therapies for this disease.

Class III β-tubulin (TUBB3) is an isotype of tubulin in normal and malignant cells that contributes to mitosis via construction of the mitotic spindle. TUBB3 is normally very highly expressed in neuronal tissues (4). There has been great interest in TUBB3 in cancer, as it correlates with resistance to therapy. TUBB3 has been shown to be highly expressed in a variety of types of cancer, including NSCLC (5), breast cancer (6), ovarian cancer (7), head and neck cancers (8) and cancers of unknown primary site (9). There is a growing body of translational and clinical literature that shows a marked link between high TUBB3 expression and resistance to the standard microtubule inhibitor (MTI) class of drugs, the taxanes (docetaxel and paclitaxel) and vinca alkaloids (vinorelbine) (10).

Resistance to MTIs via TUBB3 expression has been best described in NSCLC. In early-stage, operable disease, in patients treated with adjuvant platinum therapy plus paclitaxel, high TUBB3 expression has been shown to correlate with decreased survival (11). In addition, in advanced, inoperable disease, treatment with taxane-based therapy has been associated with poorer response rates, decreased progression-free survival (PFS) and decreased overall survival (OS) in NSCLCs that highly express TUBB3 (12–14). Furthermore, an analysis of 202 patients with advanced NSCLC revealed that high TUBB3 expression was found more often in patients with adenocarcinoma histology, large cell carcinoma histology, diagnosis at a younger age (<59 years) and higher stage disease at diagnosis (stage IV vs. III) (14,15). Based on these data, it has been suggested that high TUBB3 may correlate with more aggressive disease in advanced NSCLC. As a result, there is expanding interest in evaluating TUBB3 as both a prognostic and predictive biomarker in NSCLC and a number of other cancers.

There is little data on TUBB3 expression and its clinical significance in SCLC. One small study that evaluated TUBB3 expression in neuroendocrine tumors included an analysis of 19 primary and 20 metastatic SCLC specimens (16). This study revealed a mean labeling index (MLI) of 75% (range, 45–92%) in malignant cells of the primary lesions and 96% (range, 1–99%) in the malignant cells of the metastatic lesions. This was comparable to that which was observed in large cell neuroendocrine tumors (80% MLI), but markedly higher than that which was observed in atypical carcinoid tumors (25% MLI) and typical carcinoid tumors (3% MLI). There was no analysis to evaluate correlative clinical characteristics of the patients in this study. Based on these findings, we hypothesize that TUBB3 is highly expressed in SCLC. To test our hypothesis, viable SCLC specimens from the Department of Pathology, Minneapolis VA Healthcare System (Minneapolis, MN, USA) were analyzed for TUBB3 via immunohistochemistry (IHC). In addition, the degree of expression was compared with baseline patient characteristics and outcomes to identify factors that may correlate with the expression of TUBB3 in SCLC.

## Materials and methods

### Patients and tumor specimens

Patients with a diagnosis of small cell carcinoma were identified through the Minneapolis VA Hospital Tumor Registry. The five-year period from January 1, 2006, to December 31, 2010, was selected to ensure that adequate patient data and evaluable tumor specimens were available. Patients with a clinical and confirmed pathologic diagnosis of small cell carcinoma were included. We included those with lung primaries as well as those with extra-pulmonary small cell carcinomas (EPSCC), provided that the histology was consistent with small cell carcinoma or poorly differentiated (high grade or anaplastic) neuroendocrine carcinoma consistent with small cell carcinoma. Any patients who had mixed histology (i.e. small cell carcinoma with adenocarcinoma), a questionable or speculative diagnosis of small cell carcinoma, or inadequate tissue for staining were excluded.

Patient clinical data were accessed via the tumor registry and verified with the electronic medical record. Demographic data, including age at diagnosis, gender and race, were recorded. Smoking status was also included and was defined according to the following categories: No history of smoking, past history of smoking, and actively smoking at the time of diagnosis. As many patients had quit smoking near the diagnosis of their cancer, a patient had to have quit smoking for over one month prior to their diagnosis to be considered a past smoker.

Cancer-specific data regarding stage and disease burden at diagnosis were evaluated. The Veterans Administration Lung Study Group staging categories of limited- or extensive-stage disease were used to classify stage at diagnosis (17). Disease burden was measured by calculating the number of metastatic sites involved beyond the lung and included metastases to the lymph node, bone, brain, liver, soft tissue and other visceral sites. Information pertaining to the pathology specimen was also obtained. Biopsy site, as defined by biopsy of a lung lesion, lymph node or metastatic site, was included. The type of biopsy was also evaluated in terms of whether it was a core biopsy, cytology, bone marrow biopsy or autopsy specimen.

As this study was not performed in the context of a clinical trial, standard RECIST responses to therapy were not available (18). For this analysis, imaging responses to therapy were classified as a response (any measurable decrease in the size of tumor target lesions), stable disease (no measurable change in the size of tumor target lesions) and progressive disease (any measurable increase in the size of tumor target lesions or the development of new lesions). OS for each patient was calculated from diagnosis of their cancer to mortality from any cause. PFS was defined as the time period from diagnosis to first documentation of disease progression or mortality from any cause. Subjects were censored at the time of last follow-up if mortality or progression were not observed during the follow-up period. The study was reviewed and approved by the Institutional Review Board of Minneapolis VA Healthcare System (Minneapolis, MN, USA). All patient and tumor data were de-identified prior to analysis according to VA standards.

### Immunohistochemical staining and assessment

Formalin-fixed, paraffin-embedded (FFPE) tumor specimens were obtained from the Department of Pathology, Minneapolis VA Healthcare System. The archival FFPE slides stained with hematoxylin-eosin were reviewed by a pathologist (C.I.) to assess tumor volume and adequacy for immunostaining on both tissue biopsies and cytology cell-blocks. Sections (5-μm) were obtained from the corresponding FFPE blocks and stained using antibodies against neuronal Class B-tubulin (TUJ1) (1:400 dilution; monoclonal; cat. no. MMS-435P; Covance Inc., Princeton, NJ, USA) following the manufacturer’s instructions in a Leica Bond Max automated immunostainer (Leica Microsystems, Inc., Buffalo Grove, IL, USA). In brief, heat-induced epitope retrieval was performed with ethylenediaminetetraacetic acid-Tris base buffer (pH 8.9–9.1; Leica Microsystems, Inc.); primary antibody incubation time was 15 min, and a Bond polymer refined detection kit (Leica Bond; Leica Microsystems, Inc.) was used. Brain tissue was used as the positive control and muscle tissue was used as the negative control, as recommended in the antibody manual. Tumor staining was assessed by a trained pathologist (C.I.) with no knowledge of patient’s clinical data, under the light microscope at low- and high-power magnifications (×20–×200).

No validated expression scoring system exists for TUBB3. The scoring system in this analysis was derived from previously reported scoring systems of TUBB3 ‘positivity’ in NSCLC (12,14,15). These studies classify positivity for TUBB3 as >50% of malignant cells staining positive for TUBB3 with 2+ intensity or greater on a 0–3+ intensity scale. For the purpose of our study, we aimed to evaluate classes of expression in addition to positivity. Therefore, expression of TUBB3 was classified as the composite of distribution of malignant cells staining positive for the TUBB3 immunostain and the intensity of staining. Distribution of staining was classified as the percentage of malignant cells staining positive (range, 0–100%; 10% increments). Intensity of staining was defined as: 0, no staining; 1+, weak staining; 2+, moderate staining; and 3+, strong staining. Tumor samples were determined to have high expression of TUBB3 if >50% of malignant cells were positive with 3+ intensity. Moderate expression of TUBB3 was defined as >50% of malignant cells staining positive with 2+ intensity. Low expression of TUBB3 was defined as ≤50% of malignant cells staining positive and/or ≤2+ intensity of staining. Brain tissue served as the reference specimen for high expression (3+ intensity in 100% of cells). Muscle tissue served as the reference specimen for low expression (0 intensity in 0% of cells).

### Statistical analysis

The association between TUBB3 expression and clinical/biopsy characteristics was summarized by frequencies and percentages. Fisher’s exact test was used for formal hypothesis testing due to the small sample size. OS and PFS were summarized for high and low or moderate TUBB3 expression by Kaplan-Meier survival curves. Differences in survival were tested using Cox proportional hazards regression adjusting for initial therapy. Patients receiving only best supportive care were excluded from the analysis of OS and PFS. P-values less than 0.05 were considered to indicate a statistically significant difference. All calculations were completed using R version 2.15.1 (http://www.R-project.org/).

## Results

### Patient and sample characteristics

A total of 106 patients with small cell carcinoma were diagnosed during the study period. Of these 106 patients, a total of 66 had specimens that met our inclusion criteria for IHC staining. Patient and sample characteristics are outlined in Table I. There were no EPSCCs in our cohort, as all cases represented primary SCLCs. No recurrent small cell carcinomas were included. Regarding treatment, a total of 41 patients (62%) received standard chemotherapy (cisplatin or carboplatin in combination with etoposide or irinotecan), 12 patients (18%) received chemotherapy with concurrent radiation and 12 patients (18%) opted for best supportive care. At the time of analysis, 61 (92%) of the patients had died and 5 (8%) were still living.

### TUBB3 expression in SCLC

**Figure 1:**
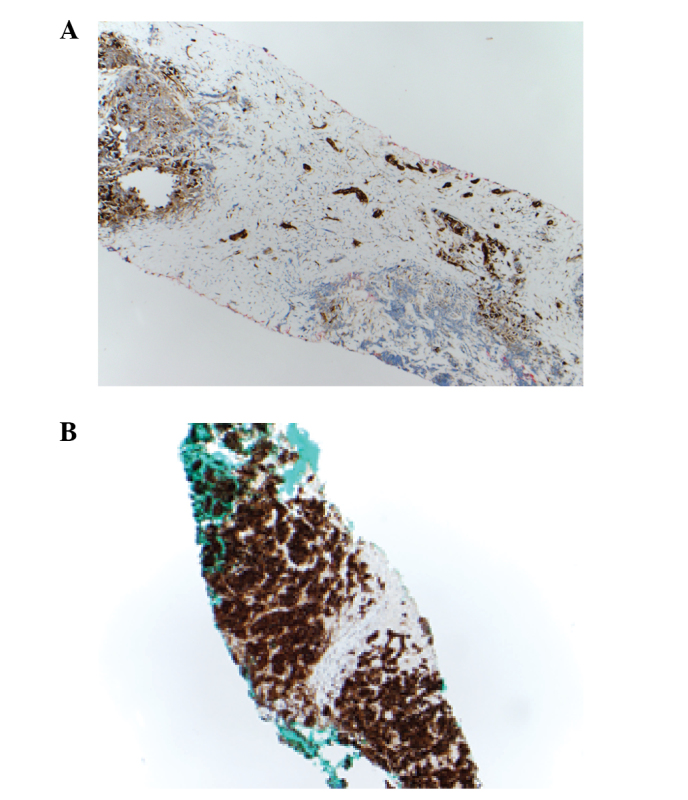
Staining pattern of class III β-tubulin in small cell lung cancer. (A) Low expression staining pattern (40% cells at 2+ intensity) and (B) high expression staining pattern (100% cells at 3+ intensity).

Of the 66 patients evaluated, 85% (95% CI, 73.9–92.5%) had high IHC expression of TUBB3. Only 4% (95% CI, 0.9–12.7%) had low TUBB3 expression and 11% (95% CI, 4.4–20.6) had moderate TUBB3 expression. The mean distribution of malignant cells staining positive for TUBB3 was also high at 87.3 ± 1.8% (mean ± SE). Based on previously reported scoring systems defined in NSCLC (12,14,15), the majority (n=63, 95%) of our specimens were positive for TUBB3. All samples had some degree of expression of TUBB3, with the lowest expression being observed in one specimen with 40% of the malignant cells staining positive at 1+ intensity. Conversely, there were 25 specimens (38%) that had 100% of the malignant cells staining positive at 3+ intensity, similar to brain tissue. Fig. 1 displays the staining pattern and intensity of a specimen with low TUBB3 expression and high TUBB3 expression.

### Correlation of TUBB3 expression with clinical and biopsy characteristics

Comparisons of TUBB3 expression (high vs. low or moderate) based on biopsy site and type of sample are outlined in Table I. Overall, there were no significant differences found among clinical characteristics including age, gender, ethnicity, smoking status, stage at diagnosis and disease burden. Additional analysis revealed that there were no significant differences in TUBB3 expression when comparing patients who presented with CNS, bone, liver or soft tissue metastases to those who did not. There was no significant difference in TUBB3 expression based on the site of the biopsy; however, there was a trend towards higher expression in the metastatic lesions biopsied. The only significant difference in TUBB3 expression was with regard to to biopsy type. Core biopsies showed significantly higher expression overall when compared to cytology specimens (P=0.004).

### TUBB3 expression and survival

Initial therapies in our cohort included standard chemotherapy (62%), chemotherapy with concurrent radiation (18%) and best supportive care (18%). Treatment and survival data were not available for one patient due to transfer of care to another institution. Data on treatment response was available for 49 of the 53 patients that received therapy. Of these, 46 (94%) responded to therapy and 3 (6%) progressed despite therapy. We were unable to complete a formal hypothesis test comparing response rate by TUBB3 expression due to the uniformly high response rate for both groups.

**Figure 2:**
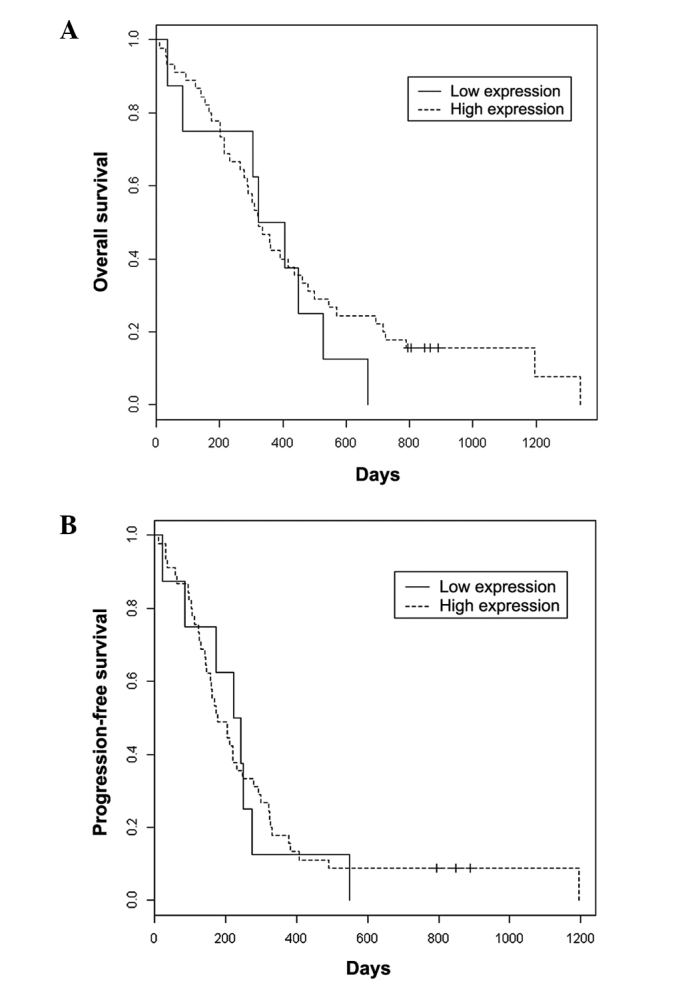
Overall and progression-free survival in treated patients. (A) Overall survival (P=0.452) and (B) progression-free survival (P=0.845).

Kaplan-Meier survival curves for OS and PFS comparing high TUBB3 expression versus low or moderate expression can be observed in Fig. 2. There was no significant difference in OS (median OS, 322 vs. 364 days; P=0.452) or PFS (median PFS, 178 vs. 233 days; P=0.845) in patients with high TUBB3 expression versus patients with low or moderate expression, respectively.

## Discussion

TUBB3 is emerging as a potential biomarker in NSCLC. Its role as a predictive and prognostic biomarker remains to be defined in a number of malignancies. In the present study, we aimed to define TUBB3 expression and correlative clinical and pathologic findings in patients with SCLC. Ultimately, we found that TUBB3 is highly expressed in SCLC, with 56 of 66 patients (85%) displaying high expression of TUBB3 in their tumors. Furthermore, using the definition of TUBB3 positivity as utilized in the NSCLC literature (12,14,15), we found that almost all of our specimens (63 of 66; 96%) were positive for TUBB3.

We were unable to identify any significant correlations between baseline clinical factors, OS or PFS in patients with high TUBB3 expression when comparing them with SCLC patients with moderate or lower expression. Other studies in NSCLC have revealed that differences in histology (adenocarcinoma and large cell carcinoma), younger age and more advanced stage at diagnosis are correlated with higher TUBB3 expression (15). Our findings do not disprove TUBB3 as a predictive biomarker in other cancers, but its role as a biomarker in SCLC may be questionable, as it has such uniformly high expression. However, the fact that TUBB3 is so highly expressed in SCLC may be one reason why the disease tends to behave more aggressively than other types of lung cancer.

In our cohort, samples obtained by core needle biopsy were more likely to have high TUBB3 expression when compared with cytology specimens. Preparation of cytology specimens into cell blocks generally has more fixation variability than that of standard core biopsies. As a result, less positive TUBB3 staining in these specimens may be observed. As TUBB3 evolves as a biomarker, the sample source should be taken into account, as variability in different sample types could lead to erroneous results. In addition, although it was not significant, there was a trend towards higher expression of TUBB3 in metastatic lesions that were biopsied. In the previous analysis of SCLC specimens, it was also noted that there was a higher percentage of positive TUBB3 staining cells in metastatic lesions (16). This raises the question of which lesions are best to sample in studies evaluating TUBB3 as a biomarker. Beyond these points, there was no other significant difference in biopsy or pathological characteristics between the high and low/moderate TUBB3 expression groups.

The high expression of TUBB3 in our cohort is markedly higher than that which has been reported in the literature for NSCLC. Numerous studies evaluating TUBB3 expression in NSCLC have defined high (or positive) expression as >50% of malignant cells staining positive (12,14,15). A review of TUBB3 expression included an analysis of three studies that utilized this 50% cut-off. This revealed that 105 of 203 (52%) cases of NSCLC specimens had high expression of TUBB3. The cut-off we selected in our study was more stringent and included staining intensity. By including only those with the highest intensity staining (3+) and using the cut-off of >50% of malignant cells staining positive, we still had 85% of our specimens with high expression. If we included those with moderate-intensity staining (2+), this number rose to 96% of our specimens. Comparing our findings to other tumors in the same review, the degree of high TUBB3 expression in our group was higher than that which has been observed in breast cancer (43% of 196 cases) and cancer of unknown primary site (55% of 40 cases) (10). Other studies using more permissive guidelines for high expression than those in our study have also revealed lower rates of high TUBB3 expression in head and neck cancer (40% of 80 cases) (8) and gastric cancer (30% of 20 cases) (19). The majority of these studies have correlated high TUBB3 expression with resistance to taxane-based therapies, and, in a number of cases, poorer outcomes.

It remains unclear why TUBB3 expression is higher in certain tumors such as NSCLC and SCLC. Normal lung tissue does not highly express TUBB3. One study evaluating tubulin isoform mRNA expression in normal and malignant tissues revealed that TUBB3 mRNA represented <5% of tubulin isoform mRNA in normal lung tissue. In the same study, the TUBB3 mRNA jumped to nearly 16% of tubulin isoform mRNA expressed in lung malignancies (20). This is much higher than that observed in normal brain tissue, where the highest expression of TUBB3 mRNA (8% of tubulin isoform mRNA) can be observed. One study in an ovarian cancer cell line revealed that hypoxia, via hypoxia-inducible factor-1a (HIF-1a), is able to induce TUBB3 expression (21). A more recent study in NSCLC showed that activating KRAS mutations correlated with a 40% increase in TUBB3 protein expression and subsequent inactivation of this pathway correlated with downregulation of TUBB3 protein expression (22). Despite these studies, there is limited knowledge of how TUBB3 is expressed in normal and malignant tissues and how it may be involved in carcinogenesis.

There were several limitations to our study. First, the number of samples viable for staining was limited. This is due to the natural history of SCLC and the fact that it is rarely treated with surgery. Therefore, even in early-stage disease, core needle biopsies or fine needle aspirates are often used for diagnosis (1). This limits the amount of stainable tissue for IHC studies. Due to the limited amount of tissue, the sample size for our secondary outcomes was limited. In addition, the scoring system for TUBB3 expression has not been clearly defined in the literature. Scoring for expression has differed among analyses, with some using only the percentage of malignant cells staining positive, and others utilizing intensity of staining as well. We selected our scoring system to fit with the literature available on NSCLC, as the data is most developed for this disease. A better method would be to use a more quantifiable method of scoring. Alternative studies have attempted to do this by quantifying TUBB3 mRNA in lieu of IHC. However, mRNA does not always correlate with the degree of protein expression (23). Therefore, the majority of studies have used IHC as the preferred measurement of TUBB3. As TUBB3 emerges as a biomarker, it is imperative that an easily reproducible and effective measurement technique is developed.

Several hypotheses can be generated from our study. The first is that high TUBB3 expression in SCLC may correlate with innate resistance to taxanes. As a single agent, paclitaxel has a response rate in the 30–50% range in chemotherapy naïve patients with SCLC (24–26). When the drug was combined with platinum agents (carboplatin and cisplatin), the overall response rates (ORRs) were 65–68% (27,28). While this ORR is comparable to the ‘gold standard’ regimen of cisplatin or carboplatin plus etoposide, the complete response (CR) rates were lower, with only ~5–10% achieving a CR with platinum plus paclitaxel versus ~20–40% achieving a CR with platinum plus etoposide (29,30). This suggests that there may be some innate resistance to taxanes in SCLC. Based on our finding of high TUBB3 expression in SCLC and the fact that this has correlated with taxane resistance in other tumors, it is possible that TUBB3 is involved in taxane resistance.

High expression of TUBB3 may make it a potential target for novel microtubule inhibitors. The epothilones are a novel class of MTIs that have been shown to be active in taxane-resistant malignancies (31). There are several taxane resistance mechanisms that this class of drug can overcome, one of which is high expression of TUBB3. In fact, the epothilones show preferential targeting of TUBB3 over other isoforms of tubulin (32). Based on the fact that our SCLC cohort showed high expression of TUBB3 throughout the majority of samples, further evaluation of this drug class in SCLC may be worthwhile.

In conclusion, our study reveals that TUBB3 is highly expressed in SCLC. This finding may elucidate why the disease is less responsive to taxane chemotherapy. In addition, TUBB3 may be a potential target for novel microtubule inhibitor therapy, such as epothilones. Further evaluation of TUBB3 as a biomarker in SCLC is warranted.

## Figures and Tables

**Table I:** Patient and sample characteristics.

Characteristics	High TUBB3 (n=56), n (%)	Low or moderate TUBB3 (n=10), n (%)	P-value
Age (years)			0.342
≤60	8 (14)	0 (0)	
>60	48 (86)	10 (100)	
Smoking status			0.492
Past smoker	24 (43)	6 (60)	
Smoker at diagnosis	32 (57)	4 (40)	
Gender			1.000
Male	56 (100)	10 (100)	
Female	0 (0)	0 (0)	
Ethnicity			1.000
Caucasian	56 (100)	7 (70)	
Other	0 (0)	3 (30)	
Stage at diagnosis			0.672
Limited	12 (21)	1 (10)	
Extensive	44 (79)	9 (90)	
Number of metastatic organ sites			1.000
≤2	33 (59)	6 (60)	
>2	23 (41)	4 (40)	
Biopsy site			0.254
Lung	32 (57)	7 (70)	
Lymph node	11 (20)	3 (30)	
Distant metastasis	13 (23)	0 (0)	
Type of sample			0.004
Core biopsy	40 (71)	2 (20)	
Cytology	14 (25)	8 (80)	
Bone marrow biopsy	1 (2)	0 (0)	
Autopsy	1 (2)	0 (0)	

TUBB3, class III β-tubulin. Limited stage is defined as disease confined to a single radiation field. Extensive stage disease is defined as disease beyond what can be included in a single radiation field (17).

## References

[1] Argiris A, Murren JR (2001). Staging and clinical prognostic factors for small-cell lung cancer. Cancer J.

[2] Chute JP, Chen T, Feigal E, Simon R, Johnson BE (1999). Twenty years of phase III trials for patients with extensive-stage small-cell lung cancer: perceptible progress. J Clin Oncol.

[3] Smyth JF (1989). Chemotherapy of small cell lung cancer. Chest.

[4] Katsetos CD, Legido A, Perentes E, Mörk SJ (2003). Class III beta-tubulin isotype: a key cytoskeletal protein at the crossroads of developmental neurobiology and tumor neuropathology. J Child Neurol.

[5] Burkhart CA, Kavallaris M, Horwitz SB (2001). The role of beta-tubulin isotypes in resistance to antimitotic drugs. Biochim Biophys Acta.

[6] Bernard-Marty C, Treilleux I, Dumontet C (2002). Microtubule-associated parameters as predictive markers of docetaxel activity in advanced breast cancer patients: results of a pilot study. Clin Breast Cancer.

[7] Ohishi Y, Oda Y, Basaki Y (2007). Expression of beta-tubulin isotypes in human primary ovarian carcinoma. Gynecol Oncol.

[8] Koh Y, Kim TM, Jeon YK (2009). Class III beta-tubulin, but not ERCC1, is a strong predictive and prognostic marker in locally advanced head and neck squamous cell carcinoma. Ann Oncol.

[9] Sève P, Reiman T, Lai R (2007). Class III beta-tubulin is a marker of paclitaxel resistance in carcinomas of unknown primary site. Cancer Chemother Pharmacol.

[10] Sève P, Dumontet C (2008). Is class III beta-tubulin a predictive factor in patients receiving tubulin-binding agents?. Lancet Oncol.

[11] Sève P, Lai R, Ding K (2007). Class III beta-tubulin expression and benefit from adjuvant cisplatin/vinorelbine chemotherapy in operable non-small cell lung cancer: analysis of NCIC JBR.10. Clin Cancer Res.

[12] Dumontet C, Isaac S, Souquet PJ (2005). Expression of class III beta tubulin in non-small cell lung cancer is correlated with resistance to taxane chemotherapy. Bull Cancer.

[13] Rosell R, Scagliotti G, Danenberg KD (2003). Transcripts in pretreatment biopsies from a three-arm randomized trial in metastatic non-small-cell lung cancer. Oncogene.

[14] Sève P, Isaac S, Trédan O (2005). Expression of class III {beta}-tubulin is predictive of patient outcome in patients with non-small cell lung cancer receiving vinorelbine-based chemotherapy. Clin Cancer Res.

[15] Sève P, Mackey J, Isaac S (2005). Class III beta-tubulin expression in tumor cells predicts response and outcome in patients with non-small cell lung cancer receiving paclitaxel. Mol Cancer Ther.

[16] Katsetos CD, Kontogeorgos G, Geddes JF (2000). Differential distribution of the neuron-associated class III beta-tubulin in neuroendocrine lung tumors. Arch Pathol Lab Med.

[17] Zelen M (1973). Keynote address on biostatistics and data retrieval. Cancer Chemother Rep 3.

[18] Therasse P, Arbuck SG, Eisenhauer EA, European Organization for Research and Treatment of Cancer, National Cancer Institute of the United States, National Cancer Institute of Canada (2000). New guidelines to evaluate the response to treatment in solid tumors. J Natl Cancer Inst.

[19] Urano N, Fujiwara Y, Doki Y (2006). Clinical significance of class III beta-tubulin expression and its predictive value for resistance to docetaxel-based chemotherapy in gastric cancer. Int J Oncol.

[20] Leandro-García LJ, Leskelä S, Landa I (2010). Tumoral and tissue-specific expression of the major human beta-tubulin isotypes. Cytoskeleton (Hoboken).

[21] Raspaglio G, Filippetti F, Prislei S (2008). Hypoxia induces class III beta-tubulin gene expression by HIF-1alpha binding to its 3′ flanking region. Gene.

[22] Levallet G, Bergot E, Antoine M (2012). High TUBB3 expression, an independent prognostic marker in patients with early non-small cell lung cancer treated by preoperative chemotherapy, is regulated by K-Ras signaling pathway. Mol Cancer Ther.

[23] Pentheroudakis G, Batistatou A, Kalogeras KT (2011). Prognostic utility of β-tubulin isotype III and correlations with other molecular and clinicopathological variables in patients with early breast cancer: a translational Hellenic Cooperative Oncology Group (HeCOG) study. Breast Cancer Res Treat.

[24] Ettinger DS, Finkelstein DM, Sarma RP, Johnson DH (1995). Phase II study of paclitaxel in patients with extensive-disease small-cell lung cancer: an Eastern Cooperative Oncology Group study. J Clin Oncol.

[25] Kirschling RJ, Grill JP, Marks RS (1999). Paclitaxel and G-CSF in previously untreated patients with extensive stage small-cell lung cancer: a phase II study of the North Central Cancer Treatment Group. Am J Clin Oncol.

[26] Graziano SL, Herndon JE, Socinski MA (2008). Phase II trial of weekly dose-dense paclitaxel in extensive-stage small cell lung cancer: cancer and leukemia group B study 39901. J Thorac Oncol.

[27] Thomas P, Castelnau O, Paillotin D (2001). Phase II trial of paclitaxel and carboplatin in metastatic small-cell lung cancer: a Groupe Français de Pneumo-Cancérologie study. J Clin Oncol.

[28] Stinchcombe TE, Mauer AM, Hodgson LD (2008). Phase II trial of paclitaxel and cisplatin in patients with extensive stage small cell lung cancer: Cancer and Leukemia Group B Trial 9430. J Thorac Oncol.

[29] Evans WK, Shepherd FA, Feld R, Osoba D, Dang P, Deboer G (1985). VP-16 and cisplatin as first-line therapy for small-cell lung cancer. J Clin Oncol.

[30] Smith IE, Evans BD, Gore ME (1987). Carboplatin (Paraplatin; JM8) and etoposide (VP-16) as first-line combination therapy for small-cell lung cancer. J Clin Oncol.

[31] Rivera E, Lee J, Davies A (2008). Clinical development of ixabepilone and other epothilones in patients with advanced solid tumors. Oncologist.

[32] Dumontet C, Jordan MA, Lee FF (2009). Ixabepilone: targeting beta III-tubulin expression in taxane-resistant malignancies. Mol Cancer Ther.

